# Experimental Comparative Analysis of the Effectiveness and Cleaning Performance of Conventional and Eco-Friendly Disinfectants Available in Romania

**DOI:** 10.3390/dj14030159

**Published:** 2026-03-11

**Authors:** Szidonia Krisztina Veress, László-István Bába, Attila Bitai, Bálint Botond Bögözi, Bernadette Kerekes-Máthé, Dániel Tamás Száva, Melinda Székely

**Affiliations:** 1 Doctoral School of Medicine and Pharmacy, Institution Organizing University Doctoral Studies-IOSUD, George Emil Palade University of Medicine, Pharmacy, Science and Technology of Targu Mures, 540142 Targu Mures, Romania; szidonia-krisztina.veress@umfst.ro; 2 Department of Oral and Maxilo-Facial Surgery, Faculty of Dentistry, George Emil Palade University of Medicine, Pharmacy, Science and Technology of Targu Mures, 38 Gheorghe Marinescu Str., 540142 Targu Mures, Romania; balint.bogozi@umfst.ro (B.B.B.); daniel.szava@umfst.ro (D.T.S.); 3 Department of Pharmacology and Clinical Pharmacy, Faculty of Pharmacy, George Emil Palade University of Medicine, Pharmacy, Science and Technology of Targu Mures, 38 Gheorghe Marinescu Str., 540142 Targu Mures, Romania; 4 Faculty of Medicine, George Emil Palade University of Medicine, Pharmacy, Science and Technology of Targu Mures, 38 Gheorghe Marinescu Str., 540142 Targu Mures, Romania; 5 Department of Teeth and Dental Arches Morphology, Faculty of Dentistry, George Emil Palade University of Medicine, Pharmacy, Science and Technology of Targu Mures, 38 Gheorghe Marinescu Str., 540142 Targu Mures, Romaniamelinda.szekely@umfst.ro (M.S.)

**Keywords:** disinfection, sustainable development, dental disinfectants, dental instruments, surgical instruments

## Abstract

**Background/Objectives**: To make a dental office more environmentally conscious, it is essential to use eco-friendly disinfectants without compromising patient safety. Our aim was to examine and compare the effectiveness of the commonly used conventional disinfectants in Romania with the effectiveness of available eco-friendly disinfectants. **Methods**: Two traditional (Gigasept, Zeta 1 Ultra), and two eco-friendly (IDactiv, Sekusept Aktiv) disinfectants and an eco-friendly (gigazyme) cleaning agent were compared. For a thorough evaluation, minimal inhibitory and bactericidal concentration (MIC/MBC) tests were conducted, along with a Bradford assay to measure the concentration of residual proteins on instruments contaminated by controlled contamination and dental office use after disinfection. **Results**: During the MIC test, with the exception of Gigazyme^®^, which is an enzymatic cleaner, all of the tested disinfectants were effective in the case of the bacterial reference strains used at the concentration prescribed by the manufacturers. After disinfection, the amount of protein remaining on the dental instruments did not exceed the maximum concentration according to the ISO 15883-5:2005 protocol, and the protein removal efficiency was between 96.04% and 99.03% for all disinfectants. In the MIC/MBC test, the peracetic acid-based Sekusept Aktiv was the most effective, while Gigazyme^®^ and BossKlein IDactiv^®^ showed the highest protein removal efficiency. **Conclusions**: The tested eco-friendly disinfectants meet the necessary performance criteria and thus offer a sustainable alternative to traditional disinfectants.

## 1. Introduction

Environmental sustainability is one of the most important issues of our time, as global warming and pollution pose a significant problem for the future of the planet [1,2]. A clean, livable environment is inextricably linked to the health of the individual. Although healthcare has a moral duty to preserve and restore health, healthcare provision unintentionally contributes significantly to environmental pollution and climate change [3,4,5]. Healthcare, and dentistry in particular, is currently unsustainable, so reducing the environmental burden is a must [5,6].

Dental practice contributes to environmental burdens primarily due to the widespread use of single-use materials and instruments, whose production and transportation are resource-intensive and generate significant waste [7]. Towards sustainability, material waste should be reduced, and the use of reusable instruments should be preferred [1,8]. Several studies have shown that reusable instruments are less harmful to the environment and more cost-effective [7]. However, reusable instruments need to be disinfected, and the use of disinfectants is one of the most significant contributors to environmental damage, mainly through ecotoxicity [4,9].

Healthcare-associated infections are the most common adverse event in healthcare [7,10,11]. This may affect not only the patients, but also the healthcare workers. In this regard, dentists are exposed to infections on a daily basis, with more than 700 types of pathogens in the oral cavity that can be spread by reusable instruments [12]. Proper disinfection not only reduces the incidence of healthcare-associated infections but also plays a role in maintaining the safe use, quality and longevity of reusable instruments [11,13,14]. Disinfectants play a crucial role in this process. These are chemical substances that can kill most pathogenic microorganisms on inanimate surfaces (except endospores), due to their active ingredients, but are less efficient compared to sterilization [15,16,17]. In addition to the active ingredients that have the desired disinfectant effect, these can also contain components such as diluents or solubilizers, surfactants, foam and pH regulators, complexing agents, and sometimes fragrances [17]. Proper disinfection and sterilization can ensure the safe use of reusable instruments and prevent the spread of infections [7,14]. The effectiveness of instrument disinfection methods should be investigated to determine the most effective options to reduce the risk of contamination in dental practice, especially in dental surgery [13].

Spaulding introduced the classification of instrument disinfection in 1957 based on the risk of transmission of infection. According to his hypothesis, it can be divided into three groups (high, intermediate, low), and healthcare workers should disinfect and sterilize the instruments and surfaces used in accordance with the risk of infection [3,16]. During dental treatments, instruments may come into contact with damaged mucosa, so high-level disinfection of instruments is required accordingly [14]. High-level disinfectants inactivate all types of microorganisms, but they are generally ineffective against a large numbers of endospores [18,19]. Glutaraldehydes, hydrogen peroxide, peracetic acid, ortho-phthalaldehyde and certain quaternary ammonium derivatives are also recognized by the Food and Drug Administration (FDA) as high-level disinfectants [7,20].

Disinfection efficacy depends on many variables, such as the active ingredient of the disinfectant, the quality and temperature of the water, and the exposure time. Efficacy can be tested according to the International Organization for Standardization (ISO) 15883-5:2005 protocol. Accordingly, the amount of protein measurable on the surface of instruments should not exceed 200 µg per instrument. Other literature recommendations suggest an endpoint of 100 µg/instrument [7]. Moreover, accepted limits may vary by region, since in the United States of America it is 6.4 µg/cm^2^ (approximately 100 µg/instrument) and in Europe it is 50–100 µg/instrument [21].

There is no clear, generally accepted definition of environmentally conscious disinfectants, but they are basically characterized by two aspects: one is that they contain substances that are rapidly biodegradable and not harmful to health or the environment, and the other is that their ingredients are of natural origin (ex. plant-based or extracted from microorganisms) [22].

In order to make dentistry more environmentally friendly, it would be advisable to use eco-friendly disinfectants without compromising patient safety. The aim of this study is to examine the effectiveness of eco-friendly disinfectants available in Romania and compare them with the disinfectants commonly used (the so-called traditional disinfectants) in dental practices, in terms of efficacy. Furthermore, the present study also aimed to assess for each disinfectant whether they inhibit bacterial growth, and correspondingly, whether they have bactericidal effects at the concentration recommended by the manufacturer for sterilization and to determine the lowest concentration at which they are still effective. Based on these objectives, the hypothesis of the study is that eco-friendly disinfectants available in Romania have antimicrobial efficacy comparable to conventional disinfectants used in dental practice for disinfection at manufacturer recommended concentrations.

## 2. Materials and Methods

In the present study, four disinfectants and one enzymatic cleaner were tested, which are listed in Table 1. Information regarding the composition and environmental awareness of the disinfectants were provided in the safety data sheets of the products. Since no universally accepted classification exists for environmentally friendly disinfectants, the environmental impact of the tested products was assessed based on manufacturer-provided information and the literature data regarding their active ingredients.

The concentration and exposure time recommended by the manufacturer for the application of the disinfectants and cleaner were followed according to the instructions for use of the products, as presented in Table 1. Information on the manufacturer-reported efficacy and chemical composition of the disinfectants is presented in Supplementary Material Table S1.

To comprehensively evaluate the effectiveness of the disinfectants, their performance was assessed in three complementary settings. First, their direct effect on bacteria was tested by assessing both minimal bactericidal concentration (MBC) and minimal inhibitory concentration (MIC) against common, highly relevant pathogens. Second, the ability of disinfectants to remove protein residues after controlled contamination with bovine serum albumin (BSA) were tested. Finally, instruments which were contaminated during treatments in the dental office were used to compare real-world performance of the disinfectants.

### 2.1. Minimal Bactericidal Concentration and Minimal Inhibitory Concentration

To evaluate the MBC and MIC of the different agents, the microbiological tests were performed in accordance with the guidelines of the European Committee for Antimicrobial Susceptibility Testing (EUCAST). Although the EUCAST principles are guidelines developed primarily for testing the effectiveness of antibiotics, their methodological principles, such as the use of control strains and standardized inoculum, can be applied to testing disinfectants as antimicrobial agents to ensure reproducibility of the results.

Dilutions of the disinfectants are listed in Table 2. The disinfectants were freshly prepared before each experiment by diluting them with distilled water according to the concentrations specified by the manufacturer. Six reference strains of microorganisms commonly associated with nosocomial infections were used (Table 3).

The inoculum was prepared containing 9990 µL of liquid Müller-Hinton medium and 10 µL of 0.5 McFarland bacterial suspension of each reference strain. For the microdilutions, microtiter plates with 8 rows and 12 wells per row were used (200 µL each). Out of the 8 rows, 6 were used, 3 rows for each disinfectant to ensure reproducibility (technical triplicates), with 2 rows left empty in between to avoid cross contamination. The disinfectants were diluted 11 times in total, achieving the concentrations presented in Table 2.

Each well contained 100 µL of inoculum and 100 µL of disinfectant in increasing dilutions. The disinfectants were prepared and diluted with distilled water. The minimal inhibitory concentration (MIC) plates were incubated for 24 h at 37 °C.

After the incubation period, the MBC test was performed. First, the wells with no visible growth were identified. Then agar-agar plates were prepared by applying a grid to the back, naming the empty wells in each cell. A 10 µL aliquot from each well without visible growth was pipetted into its designated area on the agar plate. These plates were subsequentially incubated for 12 h and examined for bacterial growth.

### 2.2. Determination of Residual Protein Quantity After Disinfection Using the Bradford Assay

For the next part of the experiment, various dental instruments (e.g., mirror, dental forceps, dental and periodontal probe, dental plugger, surgical scissors, needle forceps, elevator, tooth extraction forceps) were used. These were contaminated within the framework of controlled contamination by soaking into a solution containing bovine serum albumin (220 mL–100 µg/mL) for 30 min. In the third experiment, instruments used in the dental office during treatments were tested. In this setting the real-world performance was assessed, since the instruments came into contact with blood, saliva and pus. At this stage, tests were run in parallel, since office used and contaminated instruments were sorted separately into self-sealing bags, so that each bag contained an equal number of articulated and non-articulated instruments (five in total). This experiment was repeated five times for both settings resulting *n* = 15 different measurements (five experiments with technical triplicates).

For disinfection, 200 mL of each disinfectant was prepared per bag, diluted with distilled water at the concentration recommended by the manufacturer. After this process, the instruments were washed with distilled water, then soaked in 200 mL of ultrapure water, and 3 samples were taken from each bag. The protein concentrations of the samples were determined using a micro-Bradford method. Briefly, 500 µL of water with unknown protein concentration was combined with 500 µL Bradford reagent (Coomassie G-250 solution; Pierce™ Bradford Plus Protein Assay Reagent; Thermo Scientific, Waltham, MA USA, cat nr. 23238). Absorbance was measured at 595 nm wavelength after 10 min of incubation. A calibration curve was prepared for concentrations ranging from 2.5 to 25 µg/mL (seven distinct concentrations in total) using a molecular biology grade standard (Pierce™ Bovine Serum Albumin Standard, 2 mg/mL, Thermo Scientific, cat. nr. 23209). Concentrations were calculated using the equation of the calibration curve that was repeated each day. All curves showed good linearity with R^2^ > 0.9.

### 2.3. Statistical Analysis

The results were collected into an Excel spreadsheet and subjected to descriptive and inferential statistics using GraphPad Prism 5.0. Outliers were excluded using the Grubbs test, and normality was determined using the Kolmogorov–Smirnov test. Then, based on these, residual proteins quantity were compared using the Kruskal–Wallis test and Dunn’s post-hoc test (*n* = 15). Furthermore, the performance of the disinfectants was compared in the two experimental settings (dental office vs. controlled contamination) using the eighter, the unpaired *t* test (normally distributed data sets) or the Mann–Whitney U test (for non-Gaussian datasets). Furthermore, the amount of protein remaining after each disinfection was compared with 100 µg/instrument (contamination admitted by some recommendations, more stringent than ISO 15883-5:2005 protocol) using the one-sample *t* test and Wilcoxon signed rank test (for gaussian and non-gaussian datasets, respectively). The significance level was set at *α =* 0.05 for all statistical tests.

## 3. Results

All disinfectants except Gigazyme were able to achieve bactericidal effects at the manufacturers’ recommended concentrations, in all measurements. Gigazyme, although not a disinfectant and only an enzymatic cleaner, was able to achieve bacteriostatic effects against *Staphylococcus aureus* and *Enterococcus faecalis* even at concentrations below 2%, and was bactericidal against *Enterococcus faecalis* in 1% dilution

**Figure 1:**
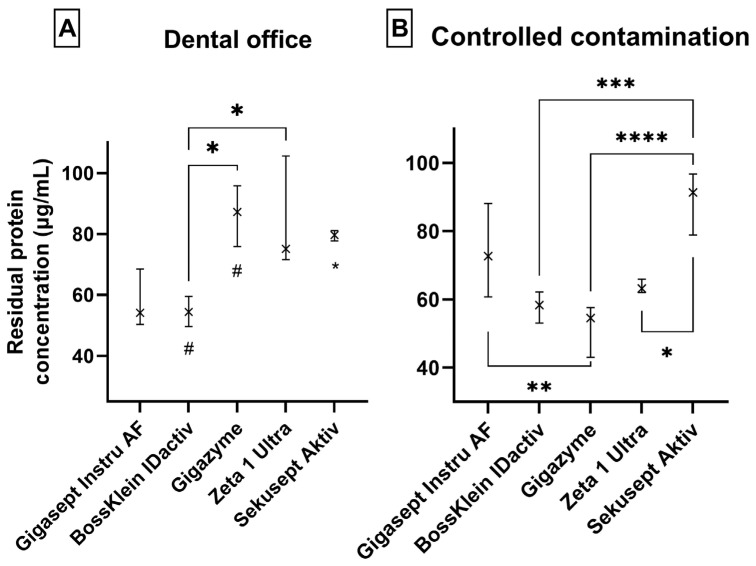
(**A**,**B**) Graphical representation of the amount of proteins remaining on the instruments after disinfection in the case of controlled contamination and instruments used in the dental office (medians [ × ] and interquartile range). * *p* < 0.05; ** *p* < 0.1; *** *p* < 0.001; **** *p* < 0.0001. (**A**) The * and # below the bars (BossKlein IDactiv^®^, Gigazime^®^ and Sekusept Aktiv^®^) represents differences between the two settings for given disinfectants (# *p* < 0.0001; * *p* < 0.05).

It can be observed in Figure 1, all the used cleaners and disinfectants were able to effectively remove proteins from the surface of the instruments, both in the case of bovine serum albumin and in the case of instruments contaminated in the dental office, thus complying with the ISO 15883-5:2005 protocol not exceeding 200 µg/instrument, but in the majority of the sets this value was even below 100 µg/instrument.

The amount of protein remaining after the use of different cleaners and disinfectants was statistically compared according to whether the instruments were contaminated with bovine serum protein or by dental office usage. The results are presented in Table 4. 

When measuring the amount of protein remaining after the disinfection of the instruments contaminated with bovine serum albumin, a statistically significant difference (*p* < 0.0001) was found in the case of the cleaner and disinfectants used. BossKlein IDactiv and Zeta 1 Ultra performed best among the disinfectants, while Gigazyme removed proteins most effectively. A difference was also found in the amount of protein remaining on instruments contaminated during dental office use before disinfection, where the BossKlein IDactiv disinfectant also had the least protein remaining on the surfaces of the instruments; however, the significance of these differences are unclear in clinical situations.

Statistically significant differences were found in the amount of protein remaining after disinfection for each disinfectant and cleaning agent compared to 100 µg/instrument (a more rigorous standard than the ISO protocol that some organizations recommend), both for instruments contaminated with bovine serum albumin and for instruments contaminated by dental office use. All disinfectants and the tested cleaner significantly reduced residual protein levels under both testing conditions (all *p* < 0.05), with effect sizes ranging from moderate to large (Cohen’s d = −0.76–−11.19; negative values indicating lower post-disinfection protein levels).

The protein removal efficiency of instruments contaminated with bovine serum albumin in terms of the amounts of proteins removed was between 97.33% and 98.77% for Gigasept instru AF^®^, between 96.66% and 99.01% for BossKlein IDactiv^®^, between 98.28% and 99.03% for Gigazyme^®^, between 98.10% and 98.54% for Zeta 1 Ultra^®^ and between 96.41% and 98.13% for Sekusept Aktiv^®^.

## 4. Discussion

Dental instruments could be contaminated with blood, secretions, and tissue remnants, and with materials used to make dental fillings and cementations, such as bonding agents, composites or cements, which can be difficult to remove. Effective cleaning is also vital to ensure microbial inactivation, as the presence of organic or inorganic residues can compromise subsequent disinfection or sterilization [10,13,32,33]. Therefore, it is important to use effective broad-spectrum cleaning and disinfection agents and to recognize the sterility of reused instruments to provide appropriate patient care [7,8,12].

The active ingredients of the disinfectants tested in the study were: (1) Quaternary ammonium compounds (QACs)—cationic surfactants bind to the negative surface of microorganisms through their permanent positive charge, inactivating energy-producing enzymes, denaturing essential cellular proteins, and disrupting the integrity of the cell membrane [15,34]; (2) Alcohol—the disinfectant effect of ethanol and isopropanol is based on the denaturation of microbial proteins, which leads to the loss of cellular functions [15]; and (3) Peracetic acid (PAA)—it exerts its effect through an oxidative mechanism: it denatures proteins, destroys the structure of the cell wall and cell membrane, oxidizes sulfhydryl and disulfide bonds, and damages cellular components by generating free radicals [18,34].

Disinfectants, whether artificial or natural, are micropollutants that always have ecological effects and have a strong impact on the ecosystem, primarily on aquatic life and potentially on soil [35,36].

Following the European CLP regulation, it is necessary to label chemical products, including disinfectants, from 1 June 2015. Substances dangerous for the environment (N) must be marked and must not be poured down the drain [17,35].

Quaternary ammonium compounds, such as benzalkonium chloride and benzethonium chloride, can be released into surface waters and sediments through wastewater treatment plants, where both small and large organisms (e.g., fish, invertebrates, aquatic plants) are directly exposed. Although their potential for bioaccumulation is low, they can persist in the environment and have ecotoxic effects on aquatic and soil microbial communities [37]. Chlorine-based disinfectants can produce halogenated estrogens, which can be detected in wastewater [38].

Peracetic acid is considered an effective yet eco-friendly disinfectant. Having a short duration of action after the disinfection process, it decomposes into water, carbon dioxide and oxygen [7,39].

Although quaternary ammonium derivatives are harmful to aquatic life, BossKlein IDactiv is labeled as an eco-friendly disinfectant. According to its manufacturer, environmental awareness is important, so they use recycled plastic in packaging and reduce the amount of plastic, and they try to replace as many ingredients as possible with plant-based ones in the composition of the disinfectants, while at the same time it does not contain alcohol or chlorides [40]. In order to more accurately examine the environmental impact of disinfectants and compare them, it would be necessary to perform a Life Cycle Assessment (LCA) analysis of the disinfectants and analyze their environmental harm within several impact categories [3,7]. Ideally, this should be done by manufacturers, but currently there is no legal obligation for them to do so, even for products labeled as environmentally conscious [5]. The concept of environmental awareness does not have a uniform definition, and sustainability claims are often difficult to verify, which contributes to the phenomenon of greenwashing [41]. Although environmentally friendly changes at the ingredient level can be considered a positive development, the true environmental impact of products can only be assessed through a comprehensive evaluation of the product as a whole.

The minimum inhibitory concentration (MIC) is the value that indicates, for a given antimicrobial agent, the lowest concentration that prevents the proliferation of microorganisms after an overnight incubation, while the minimum bactericidal concentration (MBC) represents the lowest concentration of the antimicrobial agent at which complete destruction of living bacteria occurs [42,43].

In the MBC test, an inoculum with a turbidity of 0.5 McFarland was used. According to Table 5 a difference can be observed between the dilutions of bacteriostatic and bactericidal values for some disinfectants and bacterial strains. All disinfectants, except Gigazyme, were able to achieve bactericidal effects at the manufacturer’s recommended concentrations in all measurements. Gigazyme^®^, although not a disinfectant, was able to achieve bactericidal effects even at concentrations below 2% for *Enterococcus faecalis*. The most effective disinfectant was Sekusept Aktiv^®^ (containing peracetic acid), as all bacteria—with specific differences—showed high sensitivity. It was consistently able to inhibit bacterial growth even below the manufacturer’s recommended concentrations, but the degree of sensitivity varies depending on the bacterial strain. *Enterococcus faecalis* showed the highest resistance to peracetic acid. Overall, *Staphylococcus aureus* and *S. maltophilia* showed the highest sensitivity to disinfectants. The most resistant was *Klebsiella pneumoniae*, which—except for peracetic acid—was only sensitive to disinfectants at manufacturer’s recommended concentrations.

Manufacturers’ data (available in Supplementary Table S1) and the published scientific literature show that quaternary ammonium derivatives are effective in eliminating multidrug-resistant *Staphylococcus aureus* or vancomycin-resistant *Enterococcus* [15].

A study investigating the disinfection efficacy of Bacillol^®^ (alcohol-based), Savlon^®^ (Quats and chlorhexidine-based), and Dettol^®^ (phenolic derivative) on the surfaces of striated instruments also found that all disinfectants were effective at the manufacturers’ recommended concentration after a minimum exposure time of 10 min in the case of Streptococcus mutans, Bacillus subtilis and Candida albicans [11].

After disinfection, instruments should be washed [7]. This is also important because disinfectants remaining on the surface of instruments can affect the results of protein measurements, for example, the presence of alcohol or enzymes can lead to an overestimation of protein levels [44,45].

The Bradford assay is a rapid and sensitive method for determining the amount of protein in a solution. The method is based on the binding of the dye Coomassie Blue G-250 in solution to the amino acids arginine, histidine, phenylalanine, tryptophan, and tyrosine, and on the hydrophobic interactions between the dye and the protein. The dye changes its absorbance when it binds to proteins, and its absorbance can be detected by a spectrophotometer at a wavelength of 595 nm [7,44]. Bovine serum albumin is among the most widely used carrier proteins to prepare the calibration curve [44].

The Bradford test showed that regardless of whether the instruments were contaminated with bovine serum albumin or in-office conditions, all disinfectants and cleaners used were able to effectively remove proteins from the instrument surfaces. The mean protein quantity remaining on the instrument surfaces after disinfection was between 52.684 µg and 87.895 µg for instruments contaminated with BSA, while for instruments contaminated in dental office were between 62.148 µg and 86.05 µg. This means that the disinfectants not only met the ISO 15883-5:2005 protocol, but also in majority of cases the more stringent threshold of 100 µg/instrument. Gigasept instru AF and Zeta 1 Ultra showed higher variability for instruments contaminated in the dental office; however, no statistically significant differences were found for these instruments between artificially and real-world contaminated instruments, since these disinfectants perform stably. BossKlein IDactiv, Sekusept Aktiv and Gigazyme showed statistically significant difference for artificially and real-world contaminated instruments. However, residual protein quantity (BSA—after controlled contamination) may differ in certain experimental situation, a finding that has uncertain implications yet.

Other studies have attempted to detect the amount of protein remaining on the surface of instruments after disinfection using various methods. In one study, which detected the amount of protein on the surface of endodontic files using o-phthaldialdehyde/N-acetyl cysteine, on a total of 220 files were found to have residual protein, with a mean value of 5.4 mg and values ranging from 0.5 mg to 63.2 μg [45]. Another study using to the same technique, examined the amount of residual protein after different dental cleaning methods found that 0.3–27 μg of protein residue was left on the surface of dental instruments after automated washer-disinfectors (AWD), 0.3–78 μg after manual cleaning, and 9–39 μg after manual cleaning with ultrasonic addition [46]. Our results were slightly higher (52.684–87.895 µg/instrument), but there are methodological differences, since in our experiment, the instruments were neither washed nor sonicated with the disinfectant solutions. Nevertheless, in our experiment all agents performed in accordance with the regulations.

Minor differences were found in the removal of contamination acquired in the dental office (in vivo) or bovine serum albumin (in vitro) during the disinfection of instruments. BossKlein IDactiv^®^ and Gigazyme^®^ performed better in vitro, while Sekusept Aktiv performed better under in vivo conditions. The significance of this observations is not clear yet and does not necessarily correlate with differences in terms of real-world efficiency. Indeed, considering that all disinfectants performed in accordance with the regulations, we consider these as minor differences that bear no real-world differences in terms of safety.

In the Bradford test, residual proteins were detectable in all tests, regardless of the contamination method (BSA or dental office contamination) and the disinfectant used. In all cases, statistically significant differences were found from 100 µg/instrument—the protein quantity proposed by some organizations. This observation is in-line with previous observations, since a study that measured the amount of protein remaining on the surface of endodontic files after disinfection found residual proteins in 93.3% of the instruments [15]. Another study that measured the residual protein quantity on the surface of dental hand instruments after disinfection found measurable amounts of protein in 91.2% of the cases [19]. These results highlight the fact that the disinfection process is not perfect (at least in terms of protein removal) [46]. However, the clinical significance of the residual proteins found on instruments is uncertain. Nevertheless, considering the mechanism of action of disinfectants, the protein residues lose their function and thus cannot cause infection thereafter. Regardless of this, it should be underscored that disinfection is not a substitute for sterilization [19].

According to the data available in the database “Global Antiseptic and Disinfectant Market Size & Outlook, 2024–2030”, the global antiseptics and disinfectants market shows a growing trend during the analyzed period. Among the segments, quaternary ammonium derivatives account for the largest share of the market, but market share of enzyme-based products also shows growth [47]. The data on the actual amount of disinfectants produced globally is not available in open sources, but the market size could be used as proxy for production volume. The market size was estimated at USD 5.85 billion and projected to reach USD 9.49 billion by 2030 considering a compound aggregated growth rate of 7.2% annually [46]. This information suggests that the present study addresses a relevant issue, in the context of the widespread use of disinfectants and the increasing demand for these products. The presence of substances with potential negative impact on the environment indicates the opportunity to analyze more eco-friendly alternative solutions.

Sustainability in dentistry is an important and researched topic [48]. The recognition, understanding and management of environmental concerns is slowly evolving, but is ultimately leading to a healthcare community that is more environmentally conscious [8]. The future of disinfection may lie in the development of smart bioactive and biodegradable materials, covering instrument surfaces that can inhibit biofilm adhesion, and the development of bio-derived or bioinspired antimicrobial agents. However, biosurfactants do not currently represent a potential alternative to conventional surfactants [32,36]. In Europe, disinfectants for use in the market must be approved by the European Chemicals Agency (ECHA), which, after approval of all active ingredients in the product, also assesses the environmental impact of the substance as part of the approval process [35].

Limitations of the study: The study’s limitation is that only disinfectants available in Romania were investigated. It would be necessary to study even more disinfectants to find the most effective option that is least harmful to the environment. Additional limitations are that this study only covered a limited number of bacterial strains and did not include virucidal or fungicidal evaluations. Another limitation is that, due to the lack of LCA analysis of disinfectants, the environmental impact of the tested products cannot be fully assessed; furthermore, in the absence of a unified definition of environmental awareness, it is difficult to determine which disinfectants can genuinely be considered environmentally friendly.

## 5. Conclusions

All tested disinfectants and cleaners complied with the ISO 15883-5:2005 protocol. The results indicate that eco-friendly disinfectants tested in this study are an effective alternative for disinfecting dental instruments and are less harmful to the environment. Even though we did not test all the available products that are labeled eco-friendly available on the global market, our results are promising; therefore, we encourage their use to make dental practice more environmentally friendly. Within the limitations of the applied bacteriological tests, among all the tested agents, the peracetic acid-based Sekusept Aktiv proved to be the most effective disinfectant that could represent an eco-friendly alternative to traditional disinfectants.

## Figures and Tables

**Table 1:** The disinfectants and cleaner tested in this study.

Nr	Product	Concentration	Time	Environmental Awareness	Active Ingredients	Hazard Statements
1	Gigasept instru AF^®^ (Schülke & Mayr GMBH, Norderstedt, Germany) Lot nr.: 1614201	4%	60 min	Conventional disinfectant, commonly used	Quaternary ammonium compounds, benzyl-C12-16-alkyldimethyl, chlorides Cocosalkylpropylendiamin-biguanidinium diacetat	H302 Harmful if swallowed. H314 Causes severe skin burns and eye damage. H373 May cause damage to organs (gastrointestinal tract, immune system) through prolonged or repeated exposure if swallowed. H410 Very toxic to aquatic life with long lasting effects.
2	Zeta 1 Ultra^®^ (Zhermack, Badia Polesine, Italy) Lot nr.: 448350	2%	60 min	Conventional disinfectant, commonly used	Quaternary ammonium compounds, benzyl-C12-16-alkyldimethyl, chlorides	H302 Harmful if swallowed. H314 Causes severe skin burns and eye damage. H335 May cause respiratory irritation. H373 May cause damage to organs through prolonged or repeated exposure. H410 Very toxic to aquatic life with long lasting effects.
3	IDactiv^®^ (BossKlein Topdental (Products) Ltd., Holmfield, UK) Lot nr.: 2407241	4%	60 min	Eco-friendly disinfectant	N-(3-aminopropyl)-n-dodecylpropane-1,3-diamine Poly(oxy-1,2-ethanediyl), α-[2-(didecylmethylammonio)ethyl]- ω-hydroxy-, propanoate (salt) (Bardap 26)	H314: Causes severe skin burns and eye damage. H410: Very toxic to aquatic life with long lasting effects.
4	Sekusept Aktiv^®^ (Ecolab, Saint Paul, MN, USA ) Lot nr.: 3335FM0517	3%	30 min	Eco-friendly disinfectant	Sodium Percarbonate -> peracetic acid	H302 Harmful if swallowed. H318 Risk of serious damage to eyes.
5	Gigazyme^®^ (Schülke & Mayr GMBH, Norderstedt, Germany) Lot nr.: 1626593	2%	60 min	Eco-friendly cleaner, commonly used	Protease, amylase, lipase	H319 Causes serious eye irritation

**Table 2:** Dilution of used disinfectants and cleaning agents to perform the MIC test. Concentrations are displayed in volume percents (µL/100 µL).

Dilution Step	Gigasept^®^/BossKlein IDactiv^®^	Sekusept Aktiv	Gigazyme^®^/Zeta 1 Ultra
1	4.000	3.000	2.000
2	2.000	1.500	1.000
3	1.000	0.750	0.500
4	0.500	0.375	0.250
5	0.250	0.1875	0.125
6	0.125	0.0938	0.0625
7	0.0625	0.0469	0.0313
8	0.0313	0.0234	0.0157
9	0.0156	0.0117	0.0078
10	0.0078	0.0059	0.0039
11	0.0039	0.0029	0.0020
12	0.0020	0.0015	0.0010

**Table 3:** Reference bacterial strains used in this study and their relevance.

Nr	Bacterium	ATCC Number	Relevance
1	*Escherichia coli*	*ATCC25922*	Gram-negative, facultative anaerobic rod, part of the ESKAPE pathogens and commensal flora. Its clinical relevance is manifested in opportunistic infections and ESBL (extended spectrum beta-lactamase) production [23].
2	*Klebsiella pneumoniae*	*ATCC13883*	Gram-negative, facultative anaerobic rod, part of the ESKAPE pathogens and commensal flora. It is characterized by the production of a thick polysaccharide capsule. Its clinical relevance is manifested in opportunistic infections and its high level of antibiotic resistance achieved by carbapenemase production [23,24].
3	*Enterococcus faecalis*	*ATCC29212*	Gram-positive, facultative anaerobic spherical bacterium, part of the ESKAPE pathogens and commensal flora. Vancomycin resistance makes it a very dangerous cause of hospital-acquired infections. In addition, genes associated with biocide tolerance have been described in the genus *Enterococcus* [23,25]. *E. faecalis* has been frequently implicated in failure of endodontic treatment and is also related to oral diseases, such as caries, periodontitis and peri-implantitis [26].
4	*Pseudomonas aeruginosa*	*ATCC27853*	Gram-negative, facultative anaerobic rod. Part of the ESKAPE pathogens, its clinical relevance is manifested in hospital-acquired infections, antibiotic resistance, biofilm formation and resistance to some disinfectants [23,27].
5	*Staphylococcus aureus*	*ATCC29213*	Gram-positive, facultative anaerobic, spherical skin bacterium. Part of the ESKAPE pathogens. Its clinical relevance is reflected in its high carrier rate and its resistance to antibiotics [23,28]. In the oral cavity, it may lead to a significant increase in the incidence of periodontal infections and because of its resistance to antibiotics, to the difficulty in treating them [29].
6	*Stenotrophomonas maltophilia*	*ATCC17666*	A gram-negative, strictly aerobic rod. Its clinical relevance is manifested by its biofilm formation, nosocomial infections, and high levels of innate antimicrobial resistance [30,31].

**Table 4:** Summary of the residual protein quantity after the use of Gigazyme^®^ (cleaner) and the different disinfectants. Displayed values are µg/instrument.

	Controlled Contamination (Mean ± SD)	Dental Office (Mean ± SD)	*p*-Value *
Gigasept instru AF^®^	74.57 ± 8.0	71.85 ± 33.98	0.12602
Zeta 1 Ultra^®^	63.951 ± 3.218	86.050 ± 23.985	0.357
BossKlein IDactiv^®^	60.950 ± 17.399	62.148 ± 20.145	0.00005
Sekusept Aktiv^®^	87.895 ± 8.777	79.625 ± 2.86	0.002
Gigazyme^®^	52.684 ± 9.256	84.830 ± 19.771	<0.00001

* For the Mann–Whitney U test or unpaired *t* test comparing the two settings (dental office vs. controlled contamination).

**Table 5:** Summary of the MIC test. The results are expressed in µL/mL.

MIC and MBC Values (µL/mL)	Manufacturer-Recommended Concentrations	*K. pneumoniae*		*S. maltophilia*		*E. coli*		*P. aeruginosa*		*S. aureus*		*E. faecalis*	
		MIC	MBC	MIC	MBC	MIC	MBC	MIC	MBC	MIC	MBC	MIC	MBC
Gigasept^®^	40	40	40	40	40	40	40	40	40	40	40	40	40
Zeta 1 Ultra^®^	20	20	20	10	10	10	10	0.0781	0.625	20	20	5	5
BossKlein IDactiv^®^	40	40	40	40	40	40	40	40	40	10	40	40	40
Sekusept Aktiv^®^	30	0.3125	1.875	0.1562	0.3125	0.625	30	1.25	1.875	0.312	1.25	2.5	3.75
Gigazyme^®^	20	>20	>20	>20	>20	>20	>20	>20	>20	2.5	>20	5	10

## Data Availability

Data presented are available upon request from the corresponding author.

## Supplementary Materials

The following are available online at https://www.mdpi.com/article/10.3390/dj14030159/s1.

## Abbreviations

The following abbreviations are used in this manuscript:

MIC	minimal inhibitory concentration
FDA	Food and Drug Administration
EUCAST	European Committee for Antimicrobial Susceptibility Testing
BSA	bovine serum albumin
ISO	International Organization for Standardization
AWD	automated washer-disinfector
ECHA	European Chemicals Agency
