# Supplementary material for: Experimental Comparative Analysis of the Effectiveness and Cleaning Performance of Conventional and Eco-Friendly Disinfectants Available in Romania
Source: Dent J (Basel). 2026 Mar 11;14(3):159. doi: 10.3390/dj14030159 (PMC13024747; doi:10.3390/dj14030159)
Supplement: Supplementary file 1 [file dentistry-14-00159-s001.zip › dentistry-4128711-supplementary.pdf]

Supplementary file to the manuscript entitled: Experimental comparative analysis of the effectiveness and cleaning performance of conventional and eco-friendly disinfectants available in Romania.

Dentistry journal

Authors: Szidonia Krisztina Veress [1,2], László-István Bába [3,*], Attila Bitai [4], Bálint Botond Bögözi [2], Bernadette Kerekes-Máthé [5], Dániel Tamás Száva [2] and Melinda Székely [5]

Supplementary Table S1: Manufacturer's announced efficiency based on User Manual

Nr	Product	Dilution	Action Time	Spectrum of action
**1**	Gigasept instru AF® (Schülke)	3%	15 min	Bactericidal EN 13727, EN 14561
		2%	30 min	
		1.5%	60 min	
		3%	15 min	Yeasticidal EN 13624, EN 14562
		2%	30 min	
		1.5%	60 min	
		3%	15 min	Mycobactericidal EN 14348, EN 14563
		2%	30 min	
		1.5%	60 min	
		1 %	10 min	Virucidal against enveloped viruses (incl. *HIV,HBV,HCV*) EN 14476, EN 17111
		2%	60 min	*Rotavirus* EN 14476
		4 %		*Adenovirus*
				Polyoma SV40
		3 %	5 min	ultrasonic bath (bactericidal, yeasticidal, mycobactericidal)
**2**	Zeta 1 Ultra® (Zhermack)	2% (wide disinfection)	60 min	Bactericidal EN 13727, EN 14561
				Yeasticidal EN 13624, EN 14562
				Mycobactericidal EN 14348, EN 14563
				Virucidal EN 14476 (*poliovirus, adenovirus, norovirus, HBV, HCV, HIV*)
			30 min	Ultrasonic bath: bactericidal, yeasticidal, fungicidal, mycobactericidal and virucidal
		1% (rapid disinfection, with limited	15 min	Bactericidal EN 13727, EN 14561
				Yeasticidal EN 13624, EN 14562

		spectrum of action)		Limited virucidal (*HIV, HBV, HCV, Ebola, Herpes simplex* and all human and animal influenza viruses)
	IDactiv® (BossKlein)	2%	15 min	Bactericidal<br>EN 13727, EN 14561
				Pseudomonas aeruginosa, Staphylococcus aureus, Enterococcus hirae, E. coli, Salmonella typhimurium, MRSA, Legionella pneumophilia
				Yeasticidal<br>EN 14562, EN 13624
				Enveloped viruses<br>EN 14476
				Vaccinia virus, HBV, HCV, HIV, Human T Cell Leukemia Virus, Measles Virus, HDV, Influenza virus, Coronavirus, Herpesviridae, Filoviridae (Ebola), Rubella virus, Rabies virus
		4%	30 min	Non-enveloped viruses<br>EN 17111
				Adenovirus, Norovirus
			60 min	Mycobactericidal<br>EN 14348, EN 14563
				Mycobacterium avium
				Tuberculocidal<br>EN 14348, EN 14563
				Mycobacterium terrae
**4**	Sekusept Aktiv® (Ecolab)	2%	5 min	Bactericidal<br>EN 13727, EN 14561
		1%	60 min	
		2%	5 min	Yeasticidal<br>EN 13624, EN 14562
		1%	60 min	
		4%	15 min	Fungicidal<br>EN 13624, EN 14562
		3%	30 min	
		2%	15 min	Mycobactericidal<br>EN 14348, EN 14563
		1%	60 min	
		2%	15 min	Tuberculocidal<br>EN 14348, EN 14563
		1%	60 min	
		2%	15 min	Virucidal<br>EN 14476, EN 17111
		1%	30 min	
		2%	15 min	Sporicidal<br>EN 17126
		1%		*Clostridium difficile*<br>EN 17126

Supplementary Table S2: Chemical composition of the tested disinfectants

Nr	Product	Components	CAS-No
1	Gigasept instru AF® (Schülke)	1-phenoxypropan-2-ol	770-35-4
		Cocosalkylpropylendiaminbiguanidiniumdiace- tat	Not Assigned
		Poly(oxy-1,2-ethanediyl), .alpha.-tridecyl- .omega.-hydroxy-, branched	69011-36-5
		Ethanol	64-17-5
		Amines, N-C12-14-alkyltrimethylenedi-	90640-43-0
		Quaternary ammonium compounds, benzyl- C12-16-alkyldimethyl, chlorides	68424-85-1
		Propan-2-ol	67-63-0
2	Zeta 1 Ultra® (Zhermack)	N-(3-aminopropyl)-N-d odecylpropane-1,3-dia mine	2372-82-9
		Quaternary ammonium compounds, benzyl-C12-16-alkyldi methyl, chlorides	68424-85-1
		2-aminoethanol; ethanolamine	141-43-5
		Isotridecanol, ethoxylated	69011-36-5
		Alcohols, C12-14, ethoxylatedpropoxylate d	68439-51-0
		D-glucopyrasone, oligomeric, C10-16-alkyl glycosides	110615-47-9
		N-dodecylpropane-1,3- diamine	5538-95-4
		D-Glucopyranose, oligomers, decyl octyl glycosides	68515-73-1
		Dodecylamine	124-22-1
3	IDactiv® (BossKlein)	Benzalkonium chloride	68424-85-1
		Dodecyl dimethyl ammonium chloride	7173-51-5
		Propan-2-ol	67-63-0
		Poly-hexamethylene biguanide hydrochloride	1802181-67-4
		N-(3-Aminopropyl)-N-dodecylpropane-1,3-diamine	2372-82-9
		2,2-Dimethoxy-2-phenylacetophenone	24650-42-8
		Alcohols, C9–11, Ethoxylated	68439-46-3
		Tetrasodium N,N-Bis(carboxylatomethyl)-L-glutamate	51981-21-6
		Monoethanolamine	141-43-5
		Corrosion inhibitor	Not assigned
		Cineole	470-82-6
		Hydratropaldehyde	93-53-8
		Trans-menthone	89-80-5
		P-menth-1-en-4-ol	562-74-3
		Dye	Not assigned
4	Sekusept Aktiv® (Ecolab)	Sodium Percarbonate	15630-89-4
		Citric acid	77-92-9
		Sodium carbonate	497-19-8
		solvents /additives	95-14-7
